# Thyroid hormone increases fibroblast growth factor receptor expression and disrupts cell mechanics in the developing organ of corti

**DOI:** 10.1186/1471-213X-13-6

**Published:** 2013-02-09

**Authors:** Katherine B Szarama, Núria Gavara, Ronald S Petralia, Richard S Chadwick, Matthew W Kelley

**Affiliations:** 1 Section on Developmental Neuroscience, Laboratory of Cochlear Development, National Institute on Deafness and other Communication Disorders, National Institutes of Health, Bethesda, MD, USA; 2 Section on Auditory Mechanics, Laboratory of Cellular Biology, National Institute on Deafness and other Communication Disorders, National Institutes of Health, Bethesda, MD, USA; 3 Center for Hearing and Communication Research and Department of Clinical Science, Intervention, and Technology, Karolinska Institutet, Stockholm, Sweden; 4 Advanced Imaging Core, National Institute on Deafness and other Communication Disorders, National Institutes of Health, Bethesda, MD, USA; 5 Current address: Laboratory of Joseph Opferman, Department of Biochemistry, St. Jude Children’s Research Hospital, 262 Danny Thomas Place, Memphis, TN, 38105-3678, USA; 6 Current address: Drittes Physikalisches Institut, Georg-August-Univesität, Göttingen, Germany

**Keywords:** Young’s modulus, Hair cell, Pillar cell, Hypothyroid, Cell mechanics

## Abstract

**Background:**

Thyroid hormones regulate growth and development. However, the molecular mechanisms by which thyroid hormone regulates cell structural development are not fully understood. The mammalian cochlea is an intriguing system to examine these mechanisms, as cellular structure plays a key role in tissue development, and thyroid hormone is required for the maturation of the cochlea in the first postnatal week.

**Results:**

In hypothyroid conditions, we found disruptions in sensory outer hair cell morphology and fewer microtubules in non-sensory supporting pillar cells. To test the functional consequences of these cytoskeletal defects on cell mechanics, we combined atomic force microscopy with live cell imaging. Hypothyroidism stiffened outer hair cells and supporting pillar cells, but pillar cells ultimately showed reduced cell stiffness, in part from a lack of microtubules. Analyses of changes in transcription and protein phosphorylation suggest that hypothyroidism prolonged expression of fibroblast growth factor receptors, and decreased phosphorylated Cofilin.

**Conclusions:**

These findings demonstrate that thyroid hormones may be involved in coordinating the processes that regulate cytoskeletal dynamics and suggest that manipulating thyroid hormone sensitivity might provide insight into the relationship between cytoskeletal formation and developing cell mechanical properties.

## Background

The cytoskeleton plays a key role in modulating the morphological changes of cells and tissues, impacting both the resistance to deformation and the exertion of force by the cell [1,2]. In the central nervous system, thyroid hormones are thought to act as master regulators of tissue growth and cytoskeletal development [3,4]. Thyroid hormone action can be mediated through traditional genomic mechanisms such as the regulation of transcription [5,6] in association with co-activators and co-repressors [7]. However, a direct transcriptional regulation of cytoskeletal elements by thyroid hormone receptors has not been described. This suggests that thyroid hormones might act indirectly by controlling intermediary signaling cascades, which could coordinate the regulatory components of cytoskeletal formation [8,9].

The sensory epithelium of the mammalian inner ear is an excellent model system to understand basic questions of cytoskeletal organization, as structural elements, such as actin and microtubules, facilitate the organization and architecture of this tissue. The auditory epithelium of the inner ear contains one row of inner hair cells and three rows of outer hair cells (OHCs), which detect and amplify sound, and several types of non-sensory supporting cells, which withstand the mechanical stress of sound vibrations. In particular, supporting pillar cells (PCs), which form the fluid-filled tunnel of Corti [10], have a highly organized cytoskeletal network composed primarily of microtubules [11,12]. Disruptions in the formation of this tunnel have been shown to negatively impact hearing function [13-15]. Therefore, it is imperative that we understand the regulatory cues that facilitate normal structural development.

There is reason to suggest that thyroid hormone signaling has an important role in development of the cochlea. Both the sensory and supporting cells in this epithelium have unique expression patterns of thyroid hormone receptors [16,17], and deiodinase enzymes [18,19], which control the availability of thyroid hormone. More recently, thyroid hormone transporters have also been localized to this sensory epithelium [20], and the developmental expression profile contributes some control over the accessibility of ligand to the cell-specific targets. Consistent with this hypothesis, deletion of all known thyroid hormone receptors leads to hearing impairment and inner ear cell structural defects, including malformation of cells in the organ of Corti, and a collapsed tunnel of Corti [14]. However, the factors that mediate development of the inner ear structures are largely unknown. In the cochlea, cytoskeletal formation, including the actin-based cuticular plate in OHCs [11] and the dense 15-protofilament microtubule network in PCs [21] that is mostly acetylated [12], follows cell differentiation [10-12]. However, the signaling pathways mediating cell structural development are not well understood.

Interestingly, mutations in some components of the Fibroblast growth factor (Fgf) signaling pathway cause inner ear structural defects that appear similar to those of the hypothyroid phenotype. In particular, mutations in Fgf receptor 3 (Fgfr3) lead to disruptions in tissue structure, including a collapsed tunnel of Corti, and cause auditory defects in both mice and humans [13,22-24]. These disruptions have been attributed to both a lack of differentiation of PCs and malformation in PC microtubule development [23]. However, there must also be some regulation of Fgf-receptor expression. One possibility that has been observed in other organ systems is the impact of thyroid hormone regulation of the Fgf-signaling pathway. For example, thyroid hormone stimulates Fgfr expression in undifferentiated cartilage [25,26]. Furthermore, a thyroid hormone response element is present in the promoter region of Fgfr1 [27]. Together, these data suggest that Fgf-signaling could act as an intermediary between thyroid hormone signaling and cytoskeletal development, and motivate further examination of thyroid hormone action specifically in the cochlea.

In this study, we examined the mechanism of thyroid hormone action on inner ear structural development. We found that hypothyroidism led to higher mRNA expression of Fgf-receptors relative to controls, leading to a delay in the down-regulation of Fgfr3. Hypothyroidism also led to delayed OHC and PC differentiation, which may be mediated in part by the aberrant expression of Fgf-receptors. Finally, hypothyroidism disrupted OHC and supporting PC structure, and aberrantly stiffened embryonic and early postnatal epithelial cells. Here, we show that the hypothyroid-induced cell stiffening may be mediated in part by the disrupted phosphorylation of Cofilin, which has the potential to alter actin dynamics.

## Results

### Thyroid hormone levels regulate Fgfr expression in the cochlea

**Figure 1:**
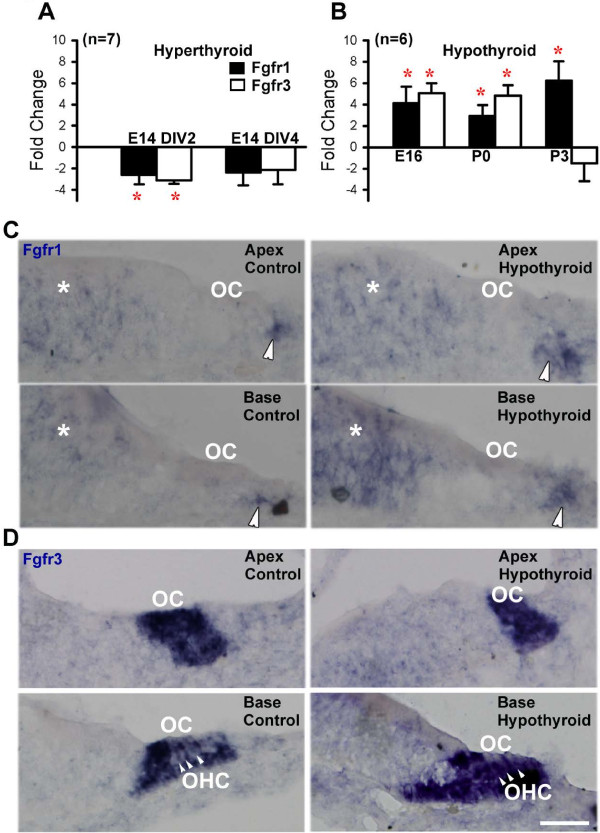
**Thyroid hormone levels regulate Fgf-receptor expression in the cochlea.** (**A**) QPCR results show significantly decreased *Fgfr1* (black) and *Fgfr3* (white) expression upon treatment with thyroid hormone *in vitro* (*p-value < 0.05). (**B**) QPCR expression of *Fgfr1* and *Fgfr3* is significantly increased when thyroid hormone levels are decreased *in vivo* (*p-value < 0.05). (**C**) *In situ* hybridization for *Fgfr1* mRNA at P0 shows increased expression in the greater (asterisk) and lesser (arrowhead) epithelial ridges in hypothyroid cochleae (right) relative to control (left). (**D**) *Fgfr3* mRNA levels at P0 persist in hypothyroid OHCs (right, arrowheads) relative to controls at the base of the cochlea (left, arrowheads). Scale bar, 20 μm, applies to all images. OC, organ of Corti; OHC, outer hair cell.

To determine if Fgf-signaling is modulated by thyroid hormones, we examined expression of two Fgf-receptors, *Fgfr1* and *Fgfr3*, in cochleae from mice with altered thyroid hormone levels. To create a hyperthyroid condition, cochlear explant cultures were established at embryonic day 14 (E14) and treated with triiodothyronine (T3) for 2 or 4 days beginning after 24 hours *in vitro*. Quantitative RT-PCR (qPCR) data showed a two-fold decrease in *Fgfr1* and *Fgfr3* mRNA levels at both time points (Figure 1A). As endogenous thyroid hormone depletion takes considerably longer than thyroid hormone stimulation in culture [28], we used pharmacological agents to deplete thyroid hormones during development *in vivo*. Pregnant female mice were rendered hypothyroid with a combination of methimazole in the drinking water and low iodine feed, which has previously been shown to induce hypothyroidism in the cochlea *in utero*[29]. To probe for changes in Fgf-receptors, mRNA was extracted from inner ear sensory epithelia. Results from qPCR indicated significant increases in both *Fgfr1* and *Fgfr3* mRNA levels under hypothyroid conditions at both embryonic day 16 (E16) and postnatal day 0 (P0) (Figure 1B). However, by postnatal day 3 (P3), while *Fgfr1* expression was six-fold greater in hypothyroid conditions relative to controls, *Fgfr3* expression was not significantly different from controls (Figure 1B). These data are consistent with negative regulation of Fgfr expression by thyroid hormones within the developing inner ear.

In order to localize the increase in expression of these receptors in the cochlea, we examined Fgfr mRNA expression by *in situ* hybridization in control and hypothyroid cochleae. At P0, *Fgfr1* is localized to cell populations in both the greater epithelial ridge, a collection of cells medial to the sensory epithelium that gives rise to the inner sulcus [30], and the lesser epithelial ridge, which is located lateral to the sensory epithelium and gives rise to the spiral ligament [31] (Figure 1C). In hypothyroid conditions, expression of *Fgfr1* appeared more intense at P0 relative to controls at basal and apical regions of the cochlear duct (Figure 1C). Expansion of the expression domain of *Fgfr1* was not observed. In contrast with *Fgfr1*, *Fgfr3* is normally initially expressed broadly within the sensory epithelium in progenitors that will give rise to both hair cells and supporting cells [32], but by P0 is down-regulated in sensory OHCs of the more mature basal region of the cochlea and maintained in non-sensory supporting pillar and Deiter’s cells (Figure 1D). At later developmental time points, *Fgfr3* expression in the apex resembles expression in the base [33]. In hypothyroid conditions, *Fgfr3* expression in the base persisted in OHCs at P0, indicating a delay in down-regulation (Figure 1D). Taken together, these results show that hypothyroidism leads to a delay in development of the inner ear, and suggest that there may also be a delay in differentiation of both OHCs and PCs at postnatal stages.

**Figure 2:**
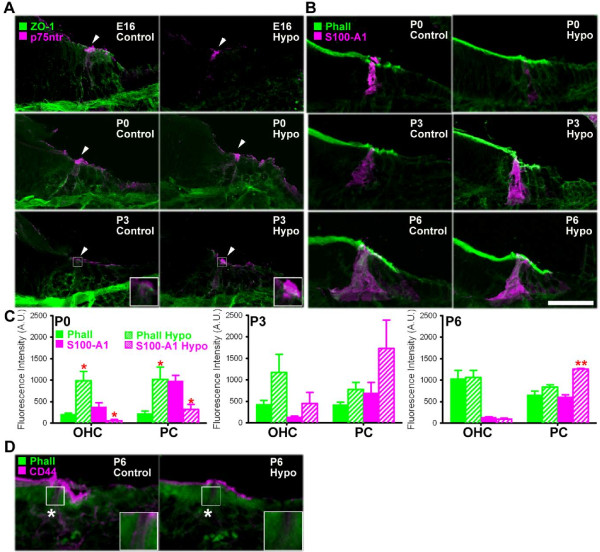
**Hypothyroidism alters supporting pillar cell development.** (**A**) Immunofluorescence for pillar cell marker p75^ntr^ (pink; arrowhead) is unchanged relative to control (left) in hypothyroid conditions (right) at E16 and P0, but persists in pillar cells (PCs) at postnatal day 3 (P3) as shown by inset (box). Tight junction protein Zonula-Occludens 1 (ZO-1) immunolabeling (green) is decreased in hypothyroid conditions at E16 and slightly decreased at P0 and P3. (**B**) Confocal micrographs of cross-sections immunolabeled for calcium-binding protein S100-A1 (pink) show increased immunofluorescence in PCs under hypothyroid conditions (right) at P3 and postnatal day 6 (P6). (**C**) Quantification of relative fluorescence intensity (mean ± s.e.m. A.U.) indicates increased phalloidin-labeling in P0 OHCs and PCs and decreased S100-A1 in OHCs and PCs from P0 hypothyroid cochleae. The increased intensity of S100-A1 persists in PCs as late as P6 (**p-value < 0.01, *p-value < 0.05). (**D**) PC marker CD44 decreases in PCs (box, inset) in hypothyroid cochleae at P6, suggesting that later differentiation of hypothyroid PCs is disrupted. Scale bar, 20 μm, applies to all confocal projections, which are representative of 6–9 samples.

As mentioned in the introduction, both gain and loss of function mutations in *Fgfr3* lead to deafness [24] and prolonged Fgf-signaling delays PC development [23,32]. Since hypothyroidism leads to a delay in down-regulation of *Fgfr3*, we hypothesized that there may be delays in the maturation of this epithelium. One hallmark of differentiation is the formation and stabilization of junctional complexes, which in the cochlea are composed of adherens and tight junction proteins [34], including Zonula-Occuludens 1 (ZO-1). In particular, ZO-1 was recently characterized in this epithelium to be associated with maturation of OHC apical structure [35]. Therefore, we examined the downstream effect of delayed maturation on immunofluorescence intensity of labeled ZO-1 in control and hypothyroid cochleae. Hypothyroid cochleae showed decreased immunolabeling of ZO-1 relative to control conditions and this was most apparent at E16 (Figure 2A), suggesting both a delay in differentiation and disruption to cell structure. To look more specifically at supporting cell maturation, expression of neurotrophin receptor p75 (p75^ntr^), an early marker of developing PCs [36], was examined by immunohistochemistry in cochlear cross-sections. P75^ntr^ is initially expressed in the cochlea at embryonic stages of development [37], and is down-regulated postnatally, which allows for specific labeling of early developing PCs. Confocal images revealed that the onset of p75^ntr^ expression was not delayed at E16 or P0 (Figure 2A). In contrast, at P3, p75^ntr^ expression in the base of the cochlea, which would normally be down-regulated, persisted in hypothyroid samples (Figure 2A), consistent with a delay in maturation of this supporting cell type. Together, these results suggest that cochlear development is delayed in the late embryonic period and PC development is delayed at early postnatal time points.

To further assess postnatal PC development, expression of S100-A1 protein was labeled and compared between cochleae in hypothyroid and control conditions. S100 proteins are initially highly enriched throughout the cochlear duct at embryonic stages [38], and are later down-regulated in differentiated PCs after the first postnatal week. While S100-A1 immunofluorescence intensity appeared to be lower in hypothyroid than in control conditions at P0, by P3, S100-A1 immunofluorescence appeared higher in hypothyroid than in control conditions (Figure 2B), which is consistent with the developmental delay observed in hypothyroid cochleae [14]. To quantify this difference, measurements of relative fluorescence intensity (mean ± s.e.m. A.U.) were calculated and compared between hypothyroid and control conditions. In PCs, S100-A1 intensity decreased from 987 ± 119 A.U. in control conditions to 319 ± 116 A.U. in hypothyroid conditions at P0, as was the case for the OHCs (Figure 2C; p-value < 0.05). Later, at P6, expression of S100-A1 was significantly increased from 609 ± 48 A.U. in controls to 1257 ± 18 A.U. in hypothyroid conditions (Figure 2C; p-value < 0.01) showing that disruptions in these cells persist at later postnatal stages. Additionally, the relative fluorescence intensity of phalloidin in hypothyroid OHCs and PCs showed about a three-fold increase relative to control conditions (Figure 2C; p-value < 0.05).

Finally, to assess late postnatal differentiation of PCs, we examined the relative immunofluorescence of CD44, a protein that is restricted to supporting PCs at P0, increases in intensity through P7 [39] and is reported to be responsive to changes in thyroid hormone levels in the cerebellum [40]. Confocal micrographs of cochlear cross-sections at P6 showed that CD44 fluorescence appeared lower in hypothyroid conditions relative to controls (Figure 2D), suggesting that PCs maintain a sensitivity to thyroid hormone levels during postnatal development that may contribute to cell maturation beyond the effects of *Fgfr3*. Taken together, these data suggest that hypothyroidism leads to a delay in the timing of PC maturation, as early markers of development that are normally down-regulated persist and late markers of development are delayed under experimental conditions. Additionally, the corresponding delay in down-regulation of Fgfr expression also suggests that some of these early effects are mediated through prolonged Fgfr expression.

### Hypothyroidism disrupts microtubule formation in the cochlea

**Figure 3:**
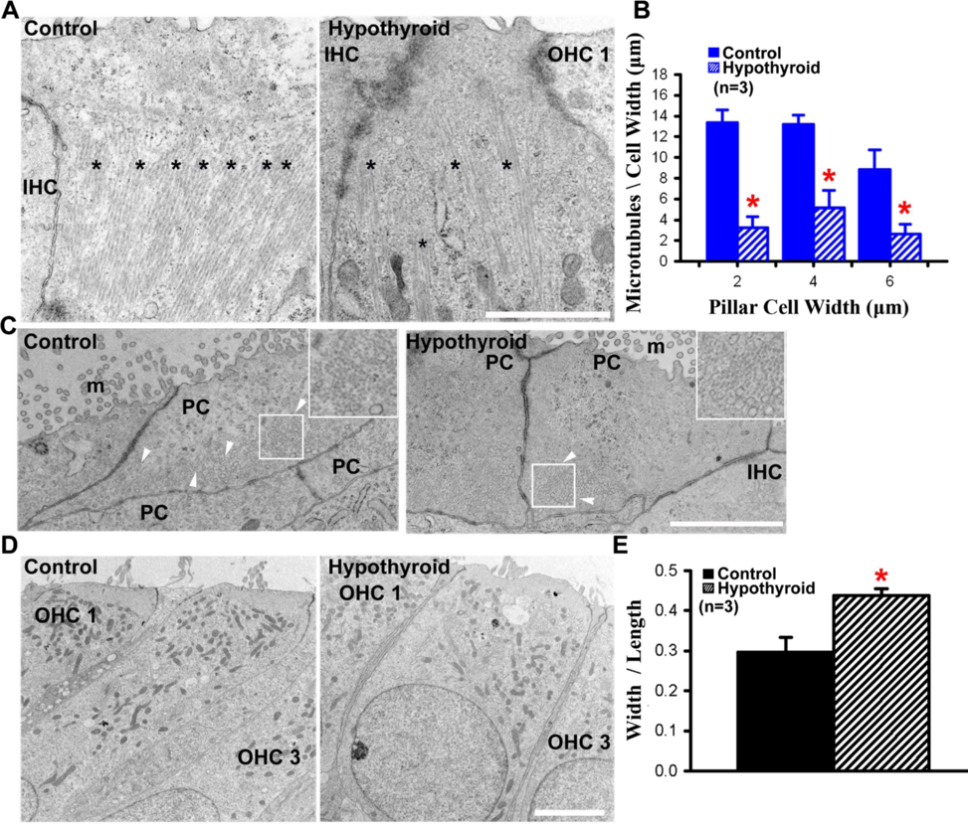
**Hypothyroidism decreases microtubule formation and alters outer hair cell morphology.** (**A**) Transmission electron micrographs through cochlear cross-sections at P3 show fewer bundles of microtubules (asterisks) in hypothyroid PCs. (**B**) Quantification indicates fewer microtubules per μm cell width at three different cell depths. (**C**) Horizontal sections representative of P3 PCs show homogeneous distribution of microtubules (insets) at the lumenal surface of control conditions (left). In hypothyroid conditions, representative horizontal sections show a decrease in total number of microtubules (right, arrowheads). (**D**) Micrographs of OHCs show that hypothyroid OHCs are shorter and wider than controls. (**E**) Quantification of the average OHC aspect ratio (mean ± s.e.m.) shows a statistically significant increase in hypothyroid conditions (*p-value < 0.001). Scale bar, 2 μm. OHC, outer hair cell; IHC, inner hair cell; PC, pillar cell; m, microvilli; P3, postnatal day 3.

Previous studies found that hypothyroidism reduces the presence of tubulin in the developing central nervous system [41,42]. Furthermore, we observed a delay in the pattern of S100-A1 expression, which has been implicated in microtubule formation *in vitro*[8,43]. Therefore we asked if hypothyroidism also had consequences for the structural development of PCs and OHCs. To examine cellular structure, transmission electron micrographs were collected from cross-sections of control and hypothyroid cochleae. At P3, control PC cytoplasm contained microtubules that are oriented longitudinally from the lumenal surface to the basilar membrane (Figure 3A). In contrast, PCs from hypothyroid cochleae contained fewer microtubules (Figure 3A). Quantitative analyses of the distribution of microtubules after controlling for differences in cell width were calculated and compared between control and hypothyroid conditions. Analysis revealed a significant decrease in the mean number of microtubules (mean ± s.e.m.) per micron cell width in PCs in hypothyroid conditions (Figure 3B; p-value < 0.05). To confirm this result, horizontal sections from the lumenal surface of the PCs were also examined and fewer microtubules were observed in hypothyroid relative to control conditions (Figure 3C). It is of note that the majority of microtubules at the lumenal cell surface of PCs in hypothyroid conditions localized to the lateral edge of the PC, whereas there were microtubules observed throughout the cytoplasm of control PCs (Figure 3C). As there was also a significant increase in hypothyroid OHC phalloidin immunofluorescence (Figure 2C), we investigated whether hypothyroid conditions would also lead to structural defects in OHC development. Transmission electron micrographs indicated that hypothyroid OHCs were shorter and wider in comparison to controls at P3 (Figure 3D). To quantify this difference, we measured the average cell width and cell length (mean ± s.e.m. μm), and found a significant increase in OHC width: length ratio in hypothyroid cochleae (Figure 3E; p-value < 0.05). Overall, the data suggest that hypothyroidism leads to microtubule-based structural defects in supporting PCs and morphological defects in sensory OHCs.

**Figure 4:**
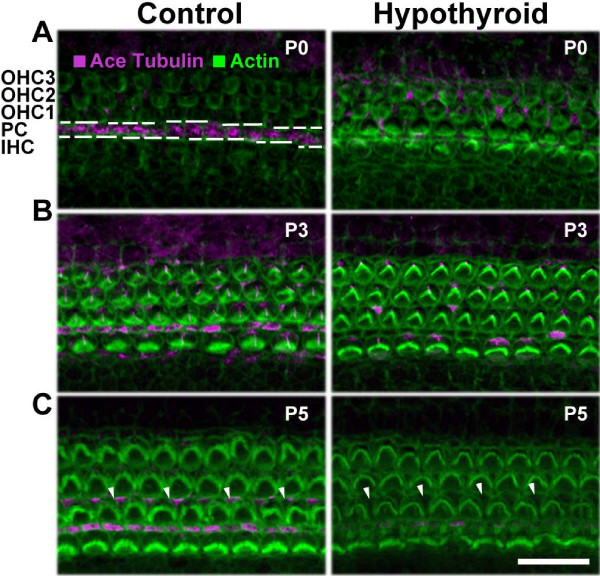
**Acetylated tubulin decreases in hypothyroid cochleae.** (**A**) Representative confocal projections of the lumenal surface of the cochlear sensory epithelium from the apex of the cochlea at postnatal day 0 (P0). Acetylated tubulin immunofluorescence (pink) is decreased in the PC row (dashed lines) of hypothyroid cochleae. Also, intensity of actin immunolabeling (green) appears increased in hypothyroid conditions. (**B**) At postnatal day 3 (P3), acetylated tubulin immunolabeling is present in the hypothyroid PC row, but is decreased relative to control. Actin immunofluorescence appears similar between control and hypothyroid conditions. (**C**) By postnatal day 5 (P5), acetylated tubulin expression appears in supporting pillar and Deiter’s cells of control cochleae, but is absent in hypothyroid conditions. Scale bar, 20 μm, applies to representative confocal projections from 12 samples. OHC, outer hair cell; PC, pillar cell; IHC, inner hair cell.

In PCs, microtubules are further refined through post-translational modifications such as acetylation [12], which have consequences for both microtubule stability [9] and cell structure [44]. To investigate the effects of hypothyroidism on microtubule stability, immunohistochemistry of acetylated tubulin was performed on whole mount cochleae from control and hypothyroid conditions. Confocal image analysis showed that acetylated tubulin immunofluorescence, a marker for stable microtubules [45,46], was almost undetectable in the PC region at P0 in hypothyroid cochleae (Figure 4A). This was in contrast to control cochleae, which showed strong immunolabeling of acetylated tubulin in PCs (Figure 4A). Similar results were observed by P3 with lowered overall fluorescence intensity levels of acetylated tubulin in hypothyroid PCs relative to control conditions (Figure 4B). OHCs at P3 also contained a microtubule-based kinocilium [47] that was identified by the enrichment of acetylated tubulin (Figure 4B). However, this structure was also observed under hypothyroid conditions (Figure 4B), suggesting that hypothyroidism does not delay formation or completely disrupt microtubules in OHCs. Finally at postnatal day 5 (P5), there was an absence of acetylated tubulin compared to control in hypothyroid PCs and Deiter’s cells (Figure 4C), a type of supporting cell with processes that interdigitate between OHCs. These results are consistent with a decrease in PC microtubule stability in response to hypothyroid conditions at all three time-points tested.

### Hypothyroidism stiffens outer hair cells and supporting pillar cells

**Figure 5:**
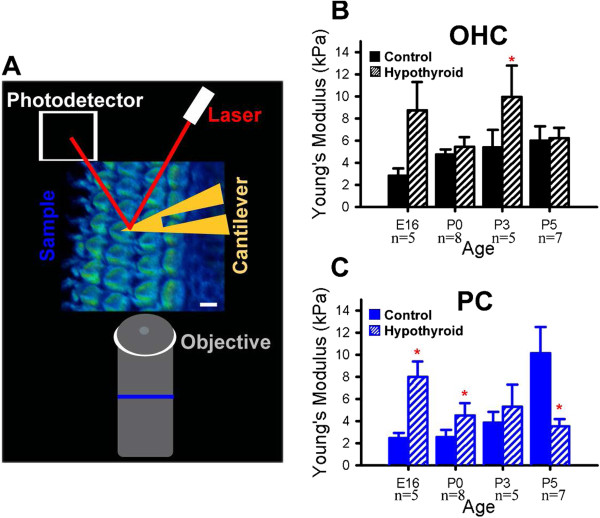
**Hypothyroidism stiffens outer hair cells and supporting pillar cells.** (**A**) Schematic of Atomic Force Microscopy (AFM) experiments. Organ of Corti explants were probed to determine surface mechanical properties of outer hair cells (OHCs) and supporting pillar cells (PCs) with an AFM that measures the relative movement of a laser reflected from a cantilever probe onto a photodiode detector. (**B**) Average Young’s modulus (mean ± s.e.m. kPa) was calculated for control and hypothyroid OHCs. OHCs in hypothyroid cochleae show a trend towards greater stiffness that is significant relative to control conditions at P3 (*p-value < 0.05). (**C**) Average Young’s modulus is significantly increased in hypothyroid PCs at E16 and P0. However, hypothyroid PCs have significantly reduced Young’s modulus by P5 relative to control conditions.

Previous cell and developmental biology studies suggest that the cytoskeleton plays a role in the control of cell shape [2] and, more recently, in dictating cell mechanical properties [1]. The decreased microtubule formation (Figure 3), and alterations in microtubule stability (Figure 4) of PCs, along with changes in morphology of OHCs (Figure 3) raised the question of whether mechanical properties of these cells might also be altered in hypothyroid conditions. To test this hypothesis, we measured the mechanical properties of these cells in culture with an atomic force microscope (Figure 5A). Average Young’s modulus (mean ± s.e.m kPa), a measure of the cellular resistance to deformation, was calculated and compared between hypothyroid and control cochleae at late embryonic and early postnatal time points. We hypothesized that decreased cell structural development would lead to a decrease in Young’s modulus. However, Young’s modulus of OHCs actually showed a trend towards increased stiffness in hypothyroid conditions between E16 and P3 (Figure 5B) with this difference becoming statistically significant at P3 (p-value < 0.05). These results suggest that hypothyroid-related changes in cell morphology stiffen OHCs.

To examine the effects of hypothyroidism on the mechanical properties of developing PCs, we also calculated average Young’s modulus for PCs in control and hypothyroid conditions. We found that in controls, PC Young’s modulus was approximately five-fold higher between E16 and P5 (Figure 5C). This corresponds to increased microtubule acetylation [48] and suggests that increased cytoskeletal formation leads to cell stiffening. In contrast with controls, hypothyroid PCs were significantly stiffer at E16 and P0 (Figure 5C; p-value < 0.05). However by P5, hypothyroid PCs showed significantly reduced Young’s modulus relative to controls (Figure 5C; p-value < 0.05). In summary, these results indicate that hypothyroidism leads to a stiffening of OHCs and PCs early in development, and that at later time points hypothyroid PCs fail to develop mature mechanical properties.

### Actin is responsible for the aberrant increase in pillar cell and outer hair cell stiffness

**Figure 6:**
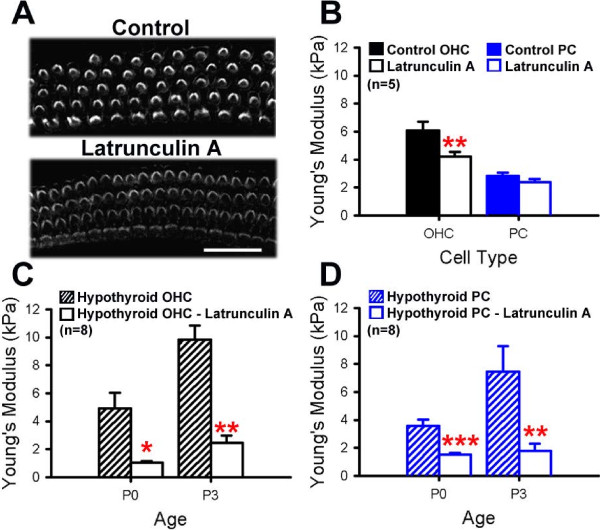
**Hypothyroidism increases sensitivity of supporting cells to actin disruption.** (**A**) Representative confocal z-projections of whole mount explants treated at P0 with Latrunculin A show decreased phalloidin immunofluorescence relative to vehicle control. (**B**) In controls, average Young’s modulus (mean ± s.e.m. kPa) of outer hair cells (OHCs, black) is significantly decreased with Latrunculin A treatment, while pillar cells (PCs, blue) are not significantly different from Latrunculin A treatment at P0 (**p-value < 0.01). (**C**) Hypothyroid OHCs also have a decreased Young’s modulus in treated conditions relative to vehicle control (*p-value < 0.05). (**D**) In contrast with controls, in which Latrunculin A treatment did not affect PC Young’s modulus, hypothyroid PCs have a significant decrease in stiffness when treated with Latrunculin A (***p-value < 0.001).

The decreased stiffness of hypothyroid PCs at P5 is consistent with the observed decrease in microtubules (Figure 3) and lack of microtubule stability (Figure 4). However, the hypothyroid PC stiffening at E16 and P0 and OHC stiffening at P3 were unexpected. One possible explanation for this effect could be the increase in filamentous actin in response to decreased thyroid hormone signaling, as observed by the increased phalloidin fluorescence intensity (Figure 2C). If actin mediates cell mechanical properties, then disruptions to the actin cytoskeleton should lead to decreased cell stiffness in hypothyroid conditions. To test this hypothesis, cochlear explants from control and hypothyroid animals were treated with Latrunculin A, a cell-permeant toxin [49] that sequesters actin monomers [50] and depolymerizes actin filaments [49]. Consistent with this mechanism, cochleae treated at P0 with Latrunculin A showed decreased phalloidin immunofluorescence (Figure 6A). In controls, a 30 minute incubation with Latrunculin A resulted in a significant decrease in the average Young’s modulus (mean ± s.e.m. kPa) of OHCs from 6.06 ± 0.64 kPa to 4.20 ± 0.31 kPa, while PCs in the same samples were not significantly different after treatment (Figure 6B; p-value < 0.01). This suggests that the disruption to OHC actin and reduced OHC stiffness does not also significantly disrupt PC Young’s modulus. However, PCs maintained in hypothyroid conditions and treated with Latrunculin A showed a significant decrease in Young’s modulus at both P0, from 4.93 ± 1.11 kPa untreated to 1.07 ± 0.08 kPa after treatment, and at P3, from 9.83 ± 1.02 kPa untreated to 2.48 ± 0.50 kPa after treatment (Figure 6D; p-value < 0.001). OHCs maintained in hypothyroid conditions also showed a decreased Young’s modulus in response to Latrunculin A administration, which is similar to results observed in euthyroid conditions. However, the magnitude of the decreased OHC Young’s modulus was much greater than in controls (Figure 6C; p-value < 0.05), suggesting a greater sensitivity to actin disruptions in hypothyroid conditions. These results suggest that PC surface mechanical properties in hypothyroid cochleae at E16 and P0 are mediated through changes in actin.

### Cofilin activity mediates changes in actin dynamics under hypothyroid conditions

**Figure 7:**
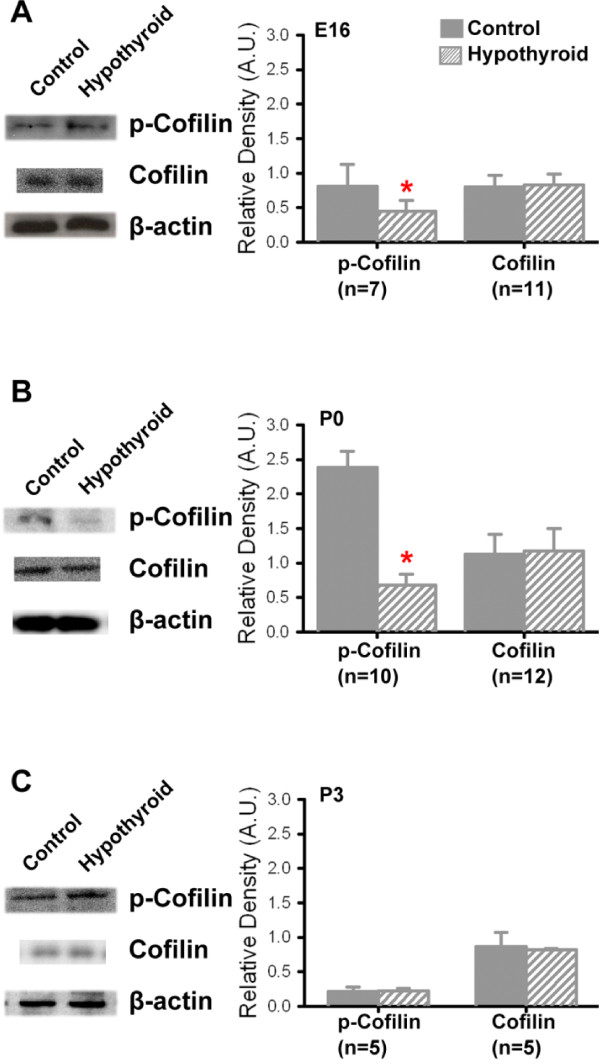
**Hypothyroidism leads to decreased phosphorylated Cofilin.** (**A**) Representative western blots for protein isolated from embryonic day 16 (E16) cochleae show decreased intensity of p-Cofilin in hypothyroid conditions relative to control. Total Cofilin appears to be unchanged. Average relative density (mean ± s.e.m.) of phosphorylated, labeled proteins indicates decreased expression of p-Cofilin in hypothyroid cochleae. Average relative density of Cofilin is not significantly different between control and hypothyroid. (**B**) At postnatal day 0 (P0), intensity of p-Cofilin in hypothyroid conditions appears to be decreased relative to control conditions. Average relative density of p-Cofilin is significantly decreased in hypothyroid relative to control conditions (*p-value < 0.05). (**C**) By postnatal day 3 (P3), the relative intensity and average relative density of p-Cofilin labeled protein is unchanged between control and treated conditions. As for E16, total protein level of Cofilin is not significantly different between control and hypothyroid cochleae at P0 and P3.

The results presented above suggested a relationship in which thyroid hormone levels might be acting through Fgfr-signaling to induce actin-based stiffening of hypothyroid OHCs and PCs. Therefore, we explored possible direct regulation of actin-modulating proteins in response to activation of the Fgf-signaling pathway. In particular, receptor tyrosine kinases have the potential to mediate actin dynamics by controlling the phosphorylation of Cofilin [51]. Cofilin, when not phosphorylated by phosphorylated-Lim-domain kinase (p-LIMK), can increase the turnover of actin leading to increased polymerization of actin filaments [52-54]. To examine the potential role of this pathway, total protein was isolated from control and hypothyroid cochleae and then probed with antibodies against phosphorylated and total Cofilin protein. At E16, hypothyroid cochleae appeared to have lower levels of p-Cofilin relative to control (Figure 7A). To quantify this difference, the average relative density (mean ± s.e.m. A.U.) of labeled protein was calculated and compared between control and hypothyroid conditions. Total Cofilin was not significantly different between control and hypothyroid conditions (Figure 7A). A similar pattern of p-Cofilin at E16 was also observed at P0, with p-Cofilin decreased from 2.38 ± 0.23 A.U. in control to 0.68 ± 0.15 A.U. in hypothyroid conditions (Figure 7B, p-value < 0.05). As was the case for E16, total protein levels of Cofilin were not significantly different between control and hypothyroid cochleae at P0 and P3. By postnatal day 3 (P3), the relative intensity and average relative density of p-Cofilin labeled proteins were now similar between control and treated conditions (Figure 7C). These results are consistent with the hypothesis that hypothyroidism leads to prolonged Fgfr expression and activation, which may have the effect of decreasing phosphorylation of Cofilin and, as a result, to increasing actin stability.

**Figure 8:**
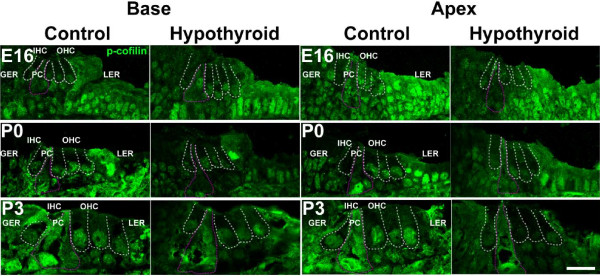
P**hosphorylated Cofilin localizes to the cochlear sensory epithelium and is decreased in hypothyroid conditions.** Representative cross-sections through the organ of Corti at the indicated time points. P-Cofilin is indicated by immunofluorescence in 12 μm projections. Immunofluorescence is present in the nuclei of cells in the greater epithelial ridge (GER) and outer hair cells (OHCs, white dashed lines), as well as in the nuclei and cytoplasm of pillar cells (PCs, pink dotted lines) and cells of the lesser epithelial ridge (LER). P-Cofilin is decreased in OHCs and PCs of hypothyroid cochleae relative to control. Scale bar, 20 μm, applies to all images. GER, greater epithelial ridge; IHC, inner hair cell; PC, pillar cell; OHC, outer hair cell; LER, lesser epithelial ridge.

While the static localization of actin filaments has long been understood to maintain cell shape and membrane tension [55,56], the dynamics of actin treadmilling for cell maturation and mechanics are just beginning to be realized. In particular, the dynamic actin cytoskeleton is primarily controlled by actin-binding proteins that regulate nucleation, polymerization and disassembly [57]. Specifically, the actin-depolymerizing factor, Cofilin, has been shown to be necessary for actin filament assembly [58,59]. The results presented above demonstrated decreased Cofilin phosphorylation during hypothyroidism. However, changes in specific cell types could not be determined. Therefore, in order to localize the observed changes in p-Cofilin in hypothyroid cochleae, the expression of p-Cofilin by immunofluorescence in cochlear cross-sections at E16, P0, and P3 was examined. At E16, expression of p-Cofilin was observed only in the nuclei of OHCs and cells of the greater epithelial ridge, but was present in both the cytoplasm and nuclei of supporting pillar cells and cells of the lesser epithelial ridge (Figure 8). This pattern of expression persisted in both basal and apical sections of control conditions through P3 (Figure 8). In hypothyroid conditions, p-Cofilin immunofluorescence appeared to be reduced in the sensory epithelium relative to controls at E16, P0, and P3 (Figure 8). In particular, a marked reduction in p-Cofilin was observed in PC cytoplasm and in OHC nuclei at P0 and P3 (Figure 8). These data support a role for lower Cofilin phosphorylation in the stabilization of actin in PCs under hypothyroid conditions. The localization of p-Cofilin in OHCs indicates that phosphorylation takes place predominantly in the nucleus, which suggests that Cofilin is active primarily in the dense actin meshwork of sensory OHCs, and may explain in part the differences in OHC and PC responses to hypothyroidism.

## Discussion

### Effects of thyroid hormone signaling in the inner ear may be regulated through prolonged Fgf-signaling

Our data support previous findings that thyroid hormone levels coordinate the timing of development in many target tissues, including the skeletal [60] and central nervous systems [61,62]. However, the specific molecular pathways that mediate the effects of thyroid hormone are largely still unknown. Thyroid hormones primarily regulate transcriptional activity through thyroid hormone receptors (reviewed in [63]). Based on similarity in phenotypes and existing data from other systems, we examined Fibroblast growth factor receptors after altering thyroid hormone levels. In the organ of Corti, decreased levels of thyroid hormone were shown to result in prolonged expression of *Fgfr1* and *Fgfr3*, respectively (Figure 1), suggesting that thyroid hormone receptors negatively regulate Fgf-receptor expression in the developing cochlea. Interestingly, *Fgfr1* has been shown to accelerate differentiation when inactivated in differentiating osteoblasts [64] and a thyroid hormone response element has been identified in the promoter region of *Fgfr1*[27]. In the organ of Corti, it seems that *Fgfr3* regulates the timing of hair cell and supporting cell development as shown by experiments in which ectopic activation of Fgfr3 delays differentiation of supporting cells [23,32]. The observation of similar delays in the down-regulation of p75^ntr^ and S100 proteins (Figure 2) in differentiating cells in hypothyroid conditions is consistent with the idea that the maintenance of Fgfr3 signaling mediates at least some of the effects observed in hypothyroidism of the cochlea. However, these data do not rule out an additional layer of regulation by thyroid hormones on Fgf-signaling. Indeed, thyroid hormone has been shown to play a role in heparin sulfate expression in the developing growth plate [65], which suggests an additional pathway, through which thyroid hormone could enhance Fgf-signaling. Overall, these findings suggest that the timing of down regulation of Fgf-signaling is important for the developing inner ear. However, the persistent expression of both Fgfr3 and of Fgf8, the most likely cognate ligand for Fgfr3 in the organ of Corti, into adult stages, suggests that Fgf-signaling may also have a continuous role in inner ear tissue morphology. While it seems reasonable to suggest that the mechanism to decrease Fgfr3-signaling would be to decrease the presence of ligand [66,67], we propose that thyroid hormones might also mediate Fgf-signaling at this stage of inner ear development by regulating the level of Fgf-receptor expression in the organ of Corti.

Using the drug methimazole, hypothyroidism was induced in utero to examine the cellular response to a lack of thyroid hormone in the organ of Corti. Thyroid hormone levels *in utero* are thought to be primarily from a maternal source and transmitted through the placenta [68,69]. Methimazole transmission has also been shown to cross the placenta [70] and to reduce the serum levels of thyroid hormone [71]. While the effectiveness of this drug has led to widespread use as a treatment in clinical cases of hyperthyroidism for adults and, in particular, during pregnancy [72], methimazole is not without side effects. In less than 1% of human subjects, agranulocytosis, a lowered white blood cell count, and liver toxicity resulting in jaundice were reported [73,74]. However, considering the relatively limited time course of methimazole treatment in these studies, it seems unlikely that the treatment led to these side effects.

### Thyroid hormone may disrupt cytoskeletal formation through prolonged Fgf-signaling

A key observation of the experiments described here was the alteration in cytoskeletal formation in response to hypothyroidism. While disruption of the cytoskeleton in cases of hypothyroidism has long been known [3,7,42], the molecular basis for these effects is not yet fully understood. The results presented here suggest that Fgf signaling may act as an intermediary, with prolonged Fgf-signaling leading to disruptions in microtubule formation. In the case of microtubule structure, we extend existing research by providing two complementary mechanisms for this hypothyroid-induced structural defect. First, deletion of *Fgfr3* leads to decreased β-tubulin [23], further supporting the relationship between Fgf-signaling and microtubule number. Second, the increase in S100-A1 in PCs is representative of elevated intracellular calcium levels [75,76], which could directly impair microtubule formation and is observed in *Fgfr3*-deficient mice [77].

In addition to the microtubule disruptions in PCs (Figures 3 and 4), there also appeared to be a higher fluorescence intensity of phalloidin-labeled actin in hypothyroid conditions relative to controls. Phalloidin, a member of the phallotoxin group of F-actin binding peptides, has been used to quantify the amount of F-actin in cells [78,79]. This probe has a diameter of 12–15 Å [80], which has the advantages of low steric hindrance to bind close to a 1:1 ratio [80] and therefore does not compete with actin binding proteins [81]. This probe also has a similar affinity for both large and small filaments [82], but does not bind monomeric G-actin. This is a limitation of the technique as it is not possible with only one probe to examine whether or not the ratio of F-actin-to-G-actin has been altered in hypothyroid relative to control conditions. However, the advantages of this probe support our conclusion that stronger phalloidin fluorescence intensity, combined with electron micrographs showing no apparent difference in cuticular plate thickness, suggests that there is a higher density of actin, which may cause in part measured stiffening at the lumenal surface of the cochlea.

There is increasing evidence that Fgf-signaling mediates actin dynamics through signaling [83], which may be the key link between hypothyroidism and the observed cell structural defects. Indeed, Fgf-signaling is necessary for the dynamic changes in actin remodeling that lead to invagination of the developing otocyst [84]. In the hypothyroid cochlea, Fgf-receptors might mediate actin dynamics through activation of p21-activated kinases (PAKs) or ROCK [85,86] that can then phosphorylate LIMK to inhibit Cofilin activity via phosphorylation [87,88]. The prolonged Fgfr-expression shown in hypothyroid conditions coincides with a decrease in phosphorylation of Cofilin (Figure 7), which is consistent with the role for this protein in the rate of actin filament turnover. Since both increased actin filaments and decreased Cofilin phosphorylation were observed in hypothyroid cochleae, these results are consistent with this proposed mode of regulation. This might in part explain the disruption to OHC morphology (Figure 3), which is interesting given that recently it has been proposed in other developing tissues that the rearrangement of actin filaments generally would produce a disruption in cell morphology [89]. Additionally, disrupted p75^ntr^ expression has also been shown to mediate actin dynamics through down-regulation of Rho-GTP signaling [90]. Since p75^ntr^ expression appeared to persist in hypothyroid conditions at P3 (Figure 2), the contribution of Fgf-signaling may also contribute indirectly through a p75^ntr^-dependent mechanism.

### Alterations in cytoskeletal dynamics may affect cell mechanical properties

Ultimately, impaired functional ability of supporting PCs to resist deformation at P5 (Figure 5) is not exactly concomitant with the decrease in microtubule number observed in electron micrographs of hypothyroid pillar cells relative to controls. However, it is possible that the lack of acetylation observed at P5 (Figure 4), and not absolute microtubule number, contributes to the formation of the tunnel of Corti by the pillar cells. It is also possible that a lack of acetylation has other effects on microtubule length and polymerization [91,92]. Future studies directly disrupting acetylation, while also controlling for microtubule length and protofilament number, will add important *in vitro* data that can be brought back into microtubule-based cell structural assays to further support what has been shown here.

In the case of actin structure, the findings that hypothyroid OHCs and PCs are aberrantly stiffer than controls (Figure 5) and are increasingly sensitive to Latrunculin A (Figure 6) implicate the actin cytoskeleton, rather than microtubule development, in hypothyroid-induced cell stiffening observed from E16 to P3. In this study, atomic force microscopy (AFM) has been used to probe cytoskeletal structures in living cells without membrane disruption or fixation. While the cell membrane is an integral part of stabilizing the cytoskeleton, previous results have shown that membrane stiffness is negligible in eukaryotic cells relative to cytoskeletal stiffness [93,94]. Furthermore, with increasing indentation depth—in this study indentation was 1.5 μm—the bulk of Young’s modulus has been shown to be the result of cytoskeletal stiffness [48], and in the case of the developing cochlea, reflects the stiffness of the actin mesh at the cell apex. Indeed, by combining AFM with live-cell fluorescence imaging techniques, it is possible to get single cell specificity. For example, after treatment with the cytoskeletal disrupting agent Latrunculin A, the OHC but not the neighboring PC Young’s modulus was decreased in cochlear explant cultures. Overall, these AFM data suggest that the calculated Young’s modulus reflects cell surface mechanical properties, which are dominated by cytoskeletal components.

### Cell-specific responses to thyroid hormone may lend insight into cell structural development

While data presented here were only able to measure the consequences of disrupted actin dynamics in hair cells and supporting PCs, disruptions to actin dynamics could have broad consequences for many supporting cell types in the developing organ of Corti. For example, the greater epithelial ridge is composed of columnar epithelial cells that undergo structural changes leading to the formation of the fluid filled space known as the inner sulcus [95]. While we know that these cells support synapse formation of sensory inner hair cells in the organ of Corti [96,97], the aberrant cell cytoplasm in this region may explain part of the pathology of hypothyroidism. By localizing phosphorylated Cofilin expression in control and hypothyroid cochlear cross-sections (Figure 8), we observed not only the aberrant defect in the sensory epithelium, but also the cell-specific differences in phosphorylated cofilin expression throughout the cochlear duct. Cofilin phosphorylation has also been observed to mediate actin dynamics in a number of developing systems [98,99]. The relationship between hypothyroidism and cell-structural defects in the developing cochlea might prove to be the basis for additional studies of morphogenesis in developing systems that rely on coordination by thyroid hormones [60,62]. Future studies also examining actin dynamics under reduced thyroid hormone levels could lend insight into a mechanism by which developmental malformations to the structure and function of the organ of Corti may be prevented.

## Conclusion

We find that hypothyroidism leads to a delay in the down-regulation of Fgf-receptors and to a decrease in microtubule formation and acetylation at early postnatal stages. While the lack of microtubules ultimately reduce supporting pillar cell stiffness, we find that hypothyroidism actually stiffens outer hair cells at P3 and pillar cells at E16 and P0 as a result of increased F-actin. Our data also show that the increased dynamics of actin in these cells might be the result of hypothyroid-induced Fgf signaling and a decreased phosphorylation of Cofilin. Together, these data implicate the sensitivity of cell structural development to thyroid hormones, and suggest that thyroid hormone signaling might coordinate the time-course of tissue morphogenesis.

## Methods

### Cochlear explant cultures and pharmacological treatments

Cochleae of Institute for Cancer Research (ICR) mice (Charles River Laboratories, Frederick, Maryland, USA) were cultured at specific stages between E16 and P5 as previously described [76] and plated on No.1 glass coverslips (Corning, New York, USA). To increase thyroid hormone signaling, explants were treated with either 5 μM triiodothyronine or 5 μM reverse triiodothyronine (Sigma, St. Louis, Missouri, USA), an inactive form of thyroid hormone, as a control, in DMSO. To decrease thyroid hormone signaling, animals were treated with 0.02% methimazole (Sigma) and 10% sucrose in drinking water and low iodine feed administered *ad libitum*. All animal care and procedures were approved by the Animal Care and Use Committee at NIH and complied with the NIH guidelines for the care and use of animals.

### Transmission electron microscopy

Cochleae from 3 control and 3 hypothyroid animals at P3 were isolated and placed immediately in 0.1 M phosphate buffer (pH 7.4) containing 4% paraformaldehyde and 2% glutaraldehyde for 30 minutes at room temperature followed by 2 hours at 4°C. The inner ears were then washed in phosphate buffer and cacodylate buffer, post-fixed with 1% osmium tetroxide, and dehydrated through a graded alcohol series before being embedded in Poly/BED 812 resin (Polysciences Inc., Warrington, Pennsylvania, USA) as previously described [48]. Thin sections of about 75 nm were cut using a Leica Reichert ultramicrotome, stained with lead citrate, mounted on 200-Cu mesh grids, and examined at room temperature using a JEOL transmission electron microscope (Akishima, Tokyo, Japan) at 80 kV with 15,000× magnification. Images were acquired using a Hamamatsu Camera (Hamamatsu Photonics K.K., Japan) and Advanced Microscopy Techniques Camera System software version 534.4 (Woburn, Massachusetts, USA). Images were cropped in Adobe Photoshop CS4 (Adobe). To quantify morphological changes in OHCs and PCs, transverse sections from three animals for each condition were analyzed with ImageJ [100] analysis software for the presence of microtubules at distances 2, 4, and 6 μm from the lumenal surface of PCs. Measurements of OHC length and width were also made. Analysis of statistical significance was determined using student’s T-test.

### Immunohistochemistry and confocal image analysis

Samples of the same time point were harvested on the same day under the same conditions, fixed in 4% paraformaldehyde for 4 hours, rinsed, passed through an increasing sucrose gradient, and embedded in OCT (Sakura, New York, USA) in parallel. All sections to be examined from the same time point for comparison were cryosectioned at 12 μm thickness on the same day. Sections were processed in parallel with the same stock solutions, which included permeabilization with 0.2% Tween-20 in PBS, blocking with 10% normal horse serum and incubation overnight in primary antibody (p75^ntr^, Covance, Princeton, New Jersey, USA, 1:1000; ZO-1, Millipore, 1:1000, S100-A1, Neomarkers, Kalamazoo, Michigan, USA, 1:500; CD44, BD Pharmigen, Franklin Lakes, New Jersey, USA, 1:200; p-Cofilin, Santa Cruz Biotechnology, Santa Cruz, California, USA, 1:500 ) at 4°C. Primary antibodies were detected using either Alexa Fluor 488 or 546 (Invitrogen, 1:1000) conjugated secondary antibodies. Directly conjugated Phalloidin 633 (Invitrogen, 1:5000) was applied to all samples. Cochleae prepared for whole mount immunohistochemistry were fixed for 2 hours and were labeled with a primary antibody against acetylated tubulin (Sigma, 1:750). Samples were mounted in Fluoromount-G (Southern Biotech, Birmingham, Alabama, USA), using No. 1Â½ glass coverslips (Corning, New York, USA) adhered with nail polish. All fluorescence images were acquired at room temperature with LSM 510 acquisition software as 12 μm Z-stacks with 1 μm optical sectioning using a Zeiss 510 LSM Confocal Microscope with 40X oil objective [1.3 numerical aperture (NA); Plan-Neofluar]. Using line scan analysis, fluorescence intensity was measured from projected Z-stacks with ImageJ [100] analysis software in 2 μm areas at the lumenal surfaces of both PCs and OHCs, as this region was previously observed with Transmission Electron Microscopy to maintain a homogeneous cell cytoplasm from E16 through P5 [48]. Average fluorescence intensity (mean ± s.e.m. Arbitrary Units) was calculated from 6 samples, and compared using student’s T-test.

### In situ hybridization

Cochleae from 6 animals per condition—hypothyroid or control—were harvested on the same day at the same time point and fixed in a stock solution of 4% paraformaldehyde in 1X PBS overnight. Samples were then rinsed, passed through an increasing sucrose gradient, and embedded in OCT (Sakura, New York, USA) in parallel using the same solutions under the same conditions. Samples were cryosectioned in parallel at 12 μm thickness. All steps of the in situ hybridization were carried out in parallel on 6 sections from 6 animals per condition as previously described [93] with probes specific to *Fgfr1* and *Fgfr3*. All samples had the same exposure to the same reaction mixture, as previously described [101] for the same length of time.

### Quantitative real-time polymerase chain reaction (qPCR)

Cochleae were removed from the temporal bone, and the surrounding mesenchyme, scala vestibuli and scala tympani were removed. 6–8 cochleae were pooled and total RNA was isolated using RNAqueous (Ambion, Grand Island, New York, USA) reagents. cDNA was synthesized from 500 ng total RNA for each condition using a Superscript III first strand synthesis kit (Invitrogen). Amplification was performed with SYBR Green (Applied Biosystems, Foster City, California, USA). Amplification of all mRNA was performed on an ABI Prism 7000 (Applied Biosystems) with the following cycling conditions: 40 cycles of 95°C for 15 seconds, and 60°C for 1 minute. To calculate fold change, gene expression was normalized to glyceraldehyde 3-phosphate dehydrogenase (GAPDH) and statistical significance was confirmed with student’s T-test. Primer sequences used are as follows: Fgfr1,F 3′-ATGGTTGACCGTTCTGGAAG-5′; Fgfr1,R 3′-AGAAAAGGGTACGCAGCAGA-5′; Fgfr3,F 3′-GAGACTTGGCTGCCAGAAAC-5′; Fgfr3, R 3′-GGGCTCACATTTGTGGTCTT-5′, GAPDH,  F  3′-ATCCTGTAGGCCAGGTCATG-5′;  GAPDH, R 3′-TATGCCCGAGGACAATAAGG-5′.

### Western blot analysis

Cochlear explants were freshly isolated before total protein extraction. Proteins were extracted from 8–10 cochleae in RIPA buffer containing complete mini protease inhibitor cocktail (Roche), complete phosphatase inhibitor cocktail (Roche), 1 mM Na_3_VO_4_, and 500 mM NaF. Protein was quantified using the Dc protein assay kit (Bio Rad) and Lowry method using a ND-1000 Spectrophotometer (Nanodrop, Wilmington, Deleware, USA). 25 μg total protein per condition was loaded onto 4-12% SDS-PAGE gels (Invitrogen) run for 2 hours at 120 V, transferred to nitrocellulose membrane (Invitrogen), and run for 3 hours at 80 V at 4°C. Membranes were blocked in 0.05% TBS-T containing BLOTTO (Rockland, Gilbertsville, Pennsylvania, USA) and 1% BSA (Sigma). Primary antibodies were incubated in blocking solution at the following concentrations: p-Cofilin (1:500, Abcam); Cofilin (1:5000, Abcam). Primary antibodies were conjugated to horseradish peroxidase anti-rabbit secondary antibody (1:5000, Amersham) and detected using ECL Detection Reagents (Amersham). Membranes were visualized using Image Station 4000R (KODAK) and Carestream Molecular Imaging Software (New Haven, Connecticut, USA). Image analysis was performed with ImageJ [100] using the Gel Analysis plug-in method to calculate relative density. Relative density of phosphorylated protein signal was normalized to β-actin (Sigma, 1:5000) signal for the same tissue and under the same experimental conditions. Statistical significance was determined with Welch’s T-test [102].

### Atomic force microscopy and live cell imaging

Experiments were performed using a Bioscope II and Bioscope Catalyst AFM (Bruker, Santa Barbara, California, USA) head mounted onto a Zeiss Axiovert 200 inverted microscope and controlled via a Nanoscope V controller. Pyramidal shaped, gold-coated, silicon nitride cantilever probes with 0.03 N/m spring constant (Bruker) were used for all measurements. Cells were identified after being loaded with 500 nM Calcein AM vital dye (Invitrogen) in Leibovitz’s media (Invitrogen) for 30 minutes and rinsed with fresh Leibovitz’s media. Contact mode AFM was applied to all samples in Leibovitz’s media at 1.5 μm maximum indentation using 3 μm ramps at 1 Hz continuous force ramping to collect 3 force-distance curves for the center of each identified hair cell or supporting cell. All force-distance curves were analyzed with custom analysis software in MATLAB (Mathworks, Natick, Massachusetts, USA) and fit to the Sneddon model [103] to measure Young’s modulus, which is a material property of the cellular resistance to deformation and was calculated with the formula F = (2/πtanα)(E/(1-ν^2^))δ^2^; where F is applied force, ν is Poisson’s ratio and assumed to be 0.5, δ is cantilever indentation, and α is cantilever tip angle. Average Young’s modulus (mean ± s.e.m. kPa) was calculated from the average of sample measurements of 10 cells within a given region of interest such as the base or the apex of the cochlea. Statistical analyses were performed using Welch’s T-test [102].

## Abbreviations

OHCs: Outer hair cells; PCs: Pillar cells; Fgf: Fibroblast growth factor; Fgfr3: Fibroblast growth factor receptor 3; P75^ntr^: Neurotrophin receptor p75; E16: Embryonic day 16; P0: Postnatal day 0; kPa: Kilo-Pascal; LIMK: Lim-domain-kinase; ROCK: Rho-associated protein kinase.

## Competing interests

The authors declare that they have no competing interests.

## Authors’ contributions

KBS designed the experiments and acquired the data, KBS and RSP performed EM experiments, NG wrote software to analyze AFM data. KBS analyzed the data and performed statistical analyses. KBS, RSP, NG, RSC, and MWK contributed to interpretation of data. KBS drafted the manuscript and RSP, NG, RSC and MWK revised it critically for important intellectual content. All authors read and approved the final manuscript.
